# Piloting I-SLEEP: a patient-centered education and empowerment intervention to improve patients’ in-hospital sleep

**DOI:** 10.1186/s40814-021-00895-z

**Published:** 2021-08-19

**Authors:** Noah R. Mason, Nicola M. Orlov, Samantha Anderson, Maxx Byron, Christine Mozer, Vineet M. Arora

**Affiliations:** 1 Pritzker School of Medicine, University of Chicago, Chicago, IL USA; 2 Department of Pediatrics, University of Chicago Medicine, Chicago, IL USA; 3 Department of Medicine, University of Chicago Medicine, 5841 S Maryland Ave, MC 2007, Chicago, IL 60637 USA

**Keywords:** Patient education, Patient empowerment, Sleep, Hospitalized patients, Nighttime disruptions

## Abstract

**Background:**

Sleep disturbances in hospitalized patients are linked to poor recovery. In preparation for a future randomized controlled trial, this pilot study evaluated the feasibility and acceptability of a multi-component intervention (I-SLEEP) that educates and empowers inpatients to advocate for fewer nighttime disruptions in order to improve sleep during periods of hospitalization.

**Methods:**

Eligible inpatients received I-SLEEP, which included an educational video, brochure, sleep kit, and three questions patients can ask their team to reduce nighttime disruptions. Following I-SLEEP, inpatients were surveyed on the primary feasibility outcomes of satisfaction with and use of I-SLEEP components. Inpatients were also surveyed regarding empowerment and understanding of intervention materials. Patient charts were reviewed to collect data on nighttime (11 PM–7 AM) vital sign and blood draws disruptions.

**Results:**

Ninety percent (*n* = 26/29) of patients were satisfied with the brochure and 87% (*n* = 27/31) with the video. Nearly all (95%, *n* = 36/37) patients felt empowered to ask their providers to minimize nighttime disruptions and 68% (*n* = 26/37) intended to alter sleep habits post-discharge. Forty-nine percent (*n* = 18/37) of patients asked an I-SLEEP question. Patients who asked an I-SLEEP question were significantly more likely to experience nights with fewer disruptions due to nighttime vitals (19% vs. 2.1%, *p* = 0.008).

**Conclusion:**

This pilot study found that I-SLEEP was well-accepted and enabled hospitalized patients to advocate for less disrupted sleep. Educating patients to advocate for reducing nighttime disruptions may be a patient-centered, low-cost strategy to improve patients’ care and in-hospital experience. These results suggest that I-SLEEP is ready to be evaluated against routine care in a future randomized controlled trial.

**Trial registration:**

ClinicalTrials.Gov NCT04151251.

## Key messages regarding feasibility

- Uncertainties regarding feasibility: This pilot study investigated the degree to which hospitalized patients would use and be satisfied with the different components of the patient-centered I-SLEEP kit. Secondarily, this study evaluated whether patients would understand the intervention materials and whether the intervention would be associated with improvements in patient empowerment and/or reductions in nighttime disruptions.
- Key feasibility findings: Nearly all participating patients were satisfied with the I-SLEEP brochure (90%) and video (87%). Well over half of the patients (63%) used at least one of the recommended I-SLEEP strategies (eye mask, ear plugs, and/or asking I-SLEEP questions to their care team) during their hospitalization. More specifically, nearly half of patients (49%) asked at least one I-SLEEP question on 51% of study nights and one quarter of patients (24%) asked all three questions during their stay. Additionally, seven of the nine patients (78%) who used the eye mask were satisfied with them.
- Implications for design of main study: Overall, the intervention was used and well-accepted by participating patients. Certain modifications to the I-SLEEP intervention—such as encouraging nurses to remind patients about the eye masks and/or altering or entirely removing the ear plugs—may further increase the intervention’s uptake.

## Background

Sleep is critical for healing, yet hospitalization is known to be disruptive to patient sleep. Environmental factors and care, such as vital signs and phlebotomy, make obtaining sufficient sleep in the hospital challenging. Inpatient sleep loss has been linked to numerous negative health outcomes, including elevated blood pressure and poor recovery [1–6].

To date, much of the research on sleep promotion has been conducted among non-hospitalized populations [6–10]. Meanwhile, the limited amount of research directed at improving sleep in the hospital has focused on staff interventions [7–9] rather than patient empowerment. In other arenas, higher patient activation and empowerment have been associated with numerous positive outcomes, including fewer unmet medical needs, increased treatment adherence, improved care experiences, and better health outcomes [10, 11]. In hospitalized patients, higher perceived control over sleep was associated with longer sleep duration, better sleep quality, and fewer reports of noise disruptions [12]. Indeed, numerous studies have found that sleep education and promotion interventions have improved sleep outcomes among diverse, non-hospitalized populations [13–17].

However, to date, no intervention has focused on the role of patient education and empowerment in reducing sleep disruptions among hospitalized patients. This pilot study evaluated the acceptability and feasibility of delivering a multi-component, patient-centered intervention (Inpatient Sleep Loss: Educating and Empowering Patients (I-SLEEP)) to educate hospitalized patients on the importance of sleep and empower them to advocate for better in-hospital sleep. The aim of this pilot study was to evaluate the acceptability and feasibility of this novel I-SLEEP intervention by examining patients’ use of and satisfaction with I-SLEEP components in a clinical practice setting. The feasibility findings from this pilot study will inform a future, full-scale randomized controlled trial comparing the effectiveness of I-SLEEP versus standard care on patient sleep and health outcomes.

## Methods

### Study design

For this single-center, pilot study of I-SLEEP, hospitalized patients were recruited from an ongoing study at the University of Chicago between July and December 2019 (Table 1) [18]. This study was approved by the University of Chicago Institutional Review Board (16685B and 19-0169). All patients provided written informed consent.

**Table 1:** Patient characteristics

Patients (*n*)	37
African American (%)	92
Female (%)	51
Age, years (mean ± SD)	52 ± 16
Highest level of education (*n*, %)	
Some high school	5 (14)
High school graduate	16 (43)
Some college or junior college	9 (24)
College graduate	2 (5)
Post-graduate	2 (5)
Length of stay, days (median, IQR)	4 (3–5)
Self-reported sleep duration in-hospital, min (mean ± SD)	388 ± 157
Prior hospitalization (%)	70
Discharge diagnosis (*n*, %)	
Blood	8 (22)
Respiratory	6 (16)
Gastrointestinal	5 (14)
Renal and urogenital	5 (14)
Skin	4 (11)
Infection	3 (8)
Cardiovascular	2 (5)
Musculoskeletal and connective tissue	2 (5)
Neurological	2 (5)
Health care empowerment (*n*, %)	
Try to get their health care providers to listen to their treatment preferences	34 (92)
Very active in their health care	33 (89)
Prefer to get as much information as possible about treatment options	33 (89)
Take their commitment to their treatment seriously	32 (86)

*SD* standard deviation, *IQR* interquartile range. Self-reported sleep duration in-hospital was measured using the Karolinska sleep log. Health care empowerment was measured using four items from the Health Care Empowerment Inventory: Informed, Committed, Collaborative, and Engaged (HCE ICCE) subscale at baseline. All other data were determined by patient chart reviews

### Patients

Hospitalized adult general medicine patients at the University of Chicago were enrolled in this pilot study. Patients were excluded from this study if they met one or more of the following criteria: (1) were admitted to the hospital longer than 72 h ago, (2) had a preexisting sleep disorder (i.e., obstructive sleep apnea, narcolepsy), (3) were transferred from the ICU or another hospital, (4) had non-contact (i.e., droplet, strict, or airborne) isolation precautions, (5) were unable to walk or on strict bedrest, (6) had cognitive or sensory deficits that would prevent them from participating in the informed consent process, or (7) were readmitted to the hospital within two weeks of their last discharge. Patient exclusion criteria were designed to exclude conditions that affect patterns of sleeping and waking [19].

### Intervention and protocol

**Fig. 1:**
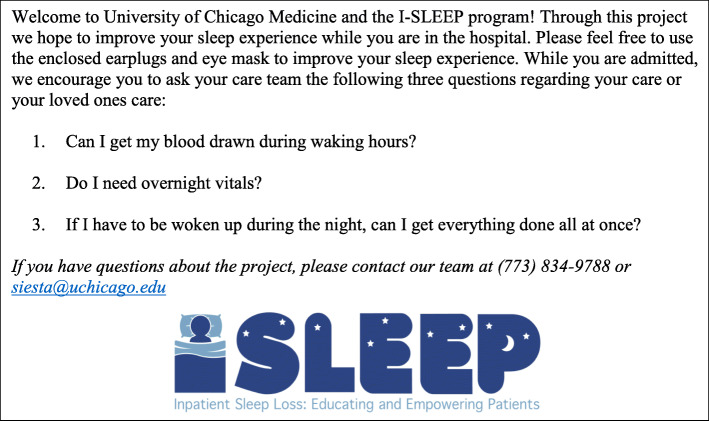
I-SLEEP notecard text. This notecard was provided to participating patients as part of the multi-component I-SLEEP intervention, along with earplugs and an eye mask. Participants were encouraged to ask their care teams the three questions below throughout their hospital stays

I-SLEEP was composed of (A) a brief patient-facing educational video, (B) an educational brochure, and (C) a sleep kit that included an eye mask, ear plugs, and a notecard highlighting three questions patients can ask their care teams to reduce nighttime disruptions in the hospital (Fig. 1). The I-SLEEP questions were the following: (1) Can I get my blood drawn during waking hours? (2) Do I need overnight vitals? (3) If I have to be woken up during the night, can I get everything done all at once? These questions were informed by a previous needs assessment among inpatients that identified vital signs and blood draws as the largest sleep disrupters [20]. The video outlined the importance of sleep, highlighted common sleep disruptions in the hospital, and shared the I-SLEEP questions. Patients viewed the video on an iPad at their bedside and were given an informational sleep brochure adapted from National Heart Lung and Blood Institute’s “Sleep Brochure” [21].

### Data collection

After I-SLEEP was delivered at patients’ bedsides, patients completed qualitative and quantitative surveys. Data was collected before the intervention and for each subsequent day during hospitalization. Patient charts were reviewed to collect nighttime (11 PM–7 AM) vital sign and blood draw disruptions [22].

### Outcomes

This study’s primary feasibility outcomes were patients’ use of the I-SLEEP components and patients’ satisfaction with the components. Success of feasibility was pre-defined as 50% or more of participating patients using at least one of the I-SLEEP components during their hospital stay and 75% or more of patients expressing satisfaction when using a component of I-SLEEP. Satisfaction with I-SLEEP components (video, brochure, eye mask, ear plugs) was measured using 5-point Likert scales. Secondarily, we also explored whether I-SLEEP was associated with improved empowerment using questions adapted from the Health Care Empowerment Inventory: Informed, Committed, Collaborative, and Engaged (HCE ICCE) and Kim Alliance scales [23, 24]. Patient understanding was measured qualitatively using the “teach-back” method [25–27]. Charts were reviewed for patient demographics and for presence of nighttime vital sign and blood draw disruptions [22].

### Data analysis

All data were collected and entered into REDCap [28], an online secure database. Descriptive statistics were used to characterize the study population and their use of I-SLEEP components. Patients’ satisfaction and empowerment Likert scores were analyzed. Patients were considered satisfied and/or empowered if their mean Likert score was ≥ 4. A qualitative thematic analysis of the open-ended “teach-back” questions evaluating patients’ understanding of I-SLEEP was conducted.

Data on vital signs and blood draw disruptions were analyzed and dichotomized into days with more nighttime (11 PM–7 AM) vital signs (≥ 2) and/or blood draws (≥ 1) versus days with more daytime and fewer nighttime vital signs (< 2) and/or blood draws (< 1). These levels were chosen based on median vital sign measurements in our hospital [20]. Chi-square tests were used to compare the percentages of nights with high versus low nighttime vital signs and blood draws between patients who asked at least one I-SLEEP question to their care team and patients who did not ask any I-SLEEP questions. Statistical significance was set at *p* < 0.05. All data were analyzed in STATA 14.0 (Stata Corp, College Station, TX).

## Results

From July to December 2019, 37 patients (Table 1) were enrolled for a combined total of 95 hospital nights. Enrolled patients were predominantly African American (92%), had prior hospitalizations (70%), were on average 52 years-old, have completed high school (43%) and/or some college or junior college (24%), and had a median hospital length of stay of four days and high levels of health care empowerment at baseline (Table 1).

While the study population generally had high levels of health care empowerment at baseline, 10 patients did report lower levels of baseline empowerment (responded neutrally or disagreed with ≥ 1 of 4 HCE ICCE empowerment questions) (Table 1). Of these 10 patients with lower empowerment at baseline, following I-SLEEP 100% (*n* = 10) agreed or strongly agreed with feeling empowered to advocate for improved sleep with their care team. Overall, 95% (*n* = 36) of patients reported feeling empowered to advocate for fewer nighttime disruptions following I-SLEEP and 68% (*n* = 26) intended to change their sleep habits post-discharge. Nearly all patients who participated in I-SLEEP were satisfied with the educational brochure (90%) and video (87%). Well over half of the patients (63%) used at least one of the recommended I-SLEEP strategies (eye mask, ear plugs, and/or asking I-SLEEP questions) during their hospitalization. Of note, the eye masks and/or ear plugs included in the I-SLEEP kit were only used by approximately one third of the patients (31%, *n* = 10). However, seven of the nine patients (78%) who wore an eye mask and one of the three patients (33%) who wore the ear plugs were satisfied with them (Table 2).

**Table 2:** Patient satisfaction and use of I-SLEEP

***Patient satisfaction with I-SLEEP intervention***^a^		
**I-SLEEP components (*n*** **= total patients)**	**% Patients satisfied (n)**	**Mean satisfaction score**^a^**(± SD)**
Educational video (*n* = 31 patients)	87 (27)	4.5 (± 0.7)
Educational brochure (*n* = 29 patients)	90 (26)	4.5 (± 0.9)
Eye mask (*n* = 9 patients)	78 (7)	3.9 (± 1.3)
Ear plugs (*n* = 3 patients)	33 (1)	2.7 (± 2.1)
***Patient use of I-SLEEP questions***^b^***(n = 37 patients; n = 95 study nights)***		
**I-SLEEP Questions**	**% Patients (n)**	**% Study Nights (n)**
Asked at least 1 question	49 (18)	51% (48)
Asked question about blood draws (Q1)	38 (14)	40% (38)
Asked question about vitals (Q2)	30 (11)	36% (34)
Asked question about batching (Q3)	38 (14)	42% (40)
Asked all 3 questions	24 (9)	28% (27)

^a^Patient satisfaction was measured on a 5-point Likert scale, from 1 (very dissatisfied) to 5 (very satisfied). Patients were considered satisfied if they scored ≥ 4 on the Likert scale. Total *n* of patients varies for different I-SLEEP components because some patients chose not to participate in different elements of the intervention (i.e., receive the brochure, wear the eye mask/earplugs)

^b^Patients were asked on a daily basis if they asked their care team any of the three provided I-SLEEP questions the night before. Question 1 was about batching nighttime disruptions: “If I have to be woken up during the night, can I get everything done all at once?” Question 2 was about blood draws: “Can I get my blood drawn during waking hours?” And question 3 was about vitals: “Do I need overnight vitals?” Data was collected from 37 patients over 95 study nights in the hospital

Half of patients (49%, *n* = 18) asked at least one I-SLEEP question on 51% of study nights (*n* = 48) (Table 2). Patients who asked just one I-SLEEP question were split between asking about reducing overnight blood draws (33%, *n* = 2), asking about reducing overnight vitals (17%, *n* = 1), and asking about getting everything done overnight at once to limit disruptions (50%, *n* = 3). One quarter (24%) of patients (*n* = 9) asked all three I-SLEEP questions during their hospital stay (Table 2). Of the 18 patients who asked at least one I-SLEEP question, 61% (*n* = 11) asked the questions to a nurse, while 11% (*n* = 2) asked a phlebotomist or certified nursing assistant and 6% (*n* = 1) asked a physician. The remaining four patients (22%) asked the questions to a combination of nurses, physicians, and/or phlebotomists.

Of the 19 patients who did not ask any of the three I-SLEEP questions, patients endorsed the following reasons for not asking the I-SLEEP questions: not remembering the questions (11%, *n* = 2), not feeling the questions applied to them (11%, *n* = 2), not having an opportunity to ask their care team the questions (21%, *n* = 4), or multiple of the previously stated reasons (21%, *n* = 4). Importantly, none endorsed feeling uncomfortable asking their care team as the reason for not asking any of the I-SLEEP questions. Patients who asked at least one but not all three I-SLEEP questions also endorsed a variety of reasons why they did not ask all three questions. The most common reason was that they did not feel all the questions applied to them. This was identified by one third of the patients (33%, *n* = 3). Only one patient (11%) endorsed not feeling comfortable asking all three questions to their care team.

While many patients understood why sleep is important and how they can improve in-hospital sleep, there were still some knowledge gaps following I-SLEEP. Half (51%, *n* = 19) of patients noted that sleep was necessary to provide energy for their daily activities, 46% (*n* = 17) stated that sleep is important for their healing and recovery, and 3% (*n* = 1) reported sleep being important for medication adherence, healthy exercise and eating habits, and/or public safety (i.e., safe driving) (Table 3). When asked about how they can improve their sleep while in the hospital, patients most frequently cited asking their care team the provided I-SLEEP questions (32%, *n* = 12) and avoiding nighttime disruptions (24%, *n* = 9) as important strategies (Table 3). Patients also mentioned that establishing a regular sleep routine (11%, *n* = 4) and increasing sleep comfort (5%, *n* = 2) were methods to improve their in-hospital sleep (Table 3).

**Table 3:** Patient understanding of I-SLEEP education (*n* = 37 patients)

Response themes	% Patients (***n***)	Representative quote
***In your own words, why is sleep important?***		
Energy for daily activities and proper functioning	51 (19)	“Sleep helps me function properly during the day.”
Healing and recovery	46 (17)	“Sleep is important because the body needs to rest, and if not, you can get sick.”
Medication adherence	3 (1)	“[Sleep is important] so I can get up and not be tired, go do my daily activities, a little bit of exercise, eat on time and take my meds.”
Proper exercise and eating habits	3 (1)	
Public safety (i.e., driving)	3 (1)	“You’ve got to make sure you get enough sleep because it might be dangerous if you don’t. If you don’t and then you go driving, you could get into an accident.”
***In your own words, how can you improve sleep in the hospital?***		
Ask care team 3 provided I-SLEEP questions	32 (12)	“I will ask [the care team] the three questions for sure and try to get everything done all at once during the night.”
Avoid nighttime disruptions	24 (9)	“I have tools now to come up with a plan and discuss moving forward how to avoid getting disturbed at night.”
Establish regular sleep routine/sleep hours	11 (4)	“Important to get rest, limit people going in and out of the room… to establish a normal sleep routine.”
Increase sleep comfort	5 (2)	“I need to get as comfortable as possible to get 8 hours of sleep.”
Not possible to improve sleep in the hospital	3 (1)	“At home I can control my sleep. Here, I’m on ‘their’ time so sleep cannot be improved here.”

Following I-SLEEP, patients were asked two open-ended “teach-back” questions to evaluate their understanding of the educational materials. Thematic analysis of patient responses was conducted

**Fig. 2:**
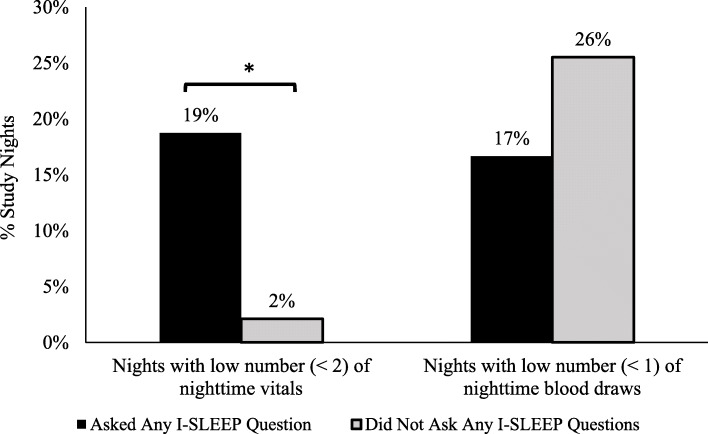
Effect of I-SLEEP questions on overnight disruptions. Overnight disruption data for vital signs and blood draws was analyzed and dichotomized into nights with high (≥ 2) versus low (< 2) numbers of vital signs or high (≥ 1) versus low (< 1) numbers of blood draws. These levels were chosen based on median vital sign measurements in the University of Chicago hospital. Asterisk indicates *p* value < 0.05

Patients who asked at least one I-SLEEP question of their providers experienced significantly more nights with fewer overnight vital sign disruptions compared to patients who did not ask any of the questions (19% vs. 2.1%, *p* = 0.008) (Fig. 2). There was no significant difference in the percentage of hospital nights with fewer nighttime blood draws by whether a patient asked an I-SLEEP question or not (17% vs. 26%, *p* = 0.29) (Fig. 2). Patients who asked the I-SLEEP question about reducing overnight vital signs were more likely to experience nights with fewer overnight vital sign disruptions as compared to patients who did not ask this question; however, this difference was not statistically significant (18% vs. 6.6%, *p* = 0.09). Similarly, patients who asked their care team about limiting overnight disruptions to one nighttime awakening were more likely to experience nights with one or fewer awakenings as compared to patients who did not ask this question, although this difference was also not statistically significant (28% vs. 16%, *p* = 0.19). There was no difference in the number of nights with fewer overnight blood draws between patients who asked the I-SLEEP question about reducing overnight blood draws and those patients who did not ask this question (18% vs. 23%, *p* = 0.6).

## Discussion

In this study, a novel inpatient education and empowerment intervention on sleep for hospitalized patients was well-received and associated with high levels of patients feeling empowered to ask their care teams about improving their in-hospital sleep. Patient feedback regarding the brochure, video, and I-SLEEP questions indicate that these materials were user-friendly and well-accepted. Furthermore, patients who asked their care team one of the I-SLEEP questions experienced nights with fewer nighttime disruptions due to vital signs. While asking at least one I-SLEEP question reduced the number of overnight vital sign disruptions, it is worth noting that it did not significantly reduce the number of nighttime blood draws. This difference in effect may be attributable to the fact that the majority of the questions were asked of nurses, who have more autonomy to change and/or forego overnight vital sign checks as opposed to blood draws which must be rescheduled by a physician order. This is consistent with previous findings at our institution which have demonstrated that there is reluctance among staff members to alter the schedule of lab orders. As such, we have found that electronic health record modifications are necessary to promote sleep-friendly lab timing [7, 29].

It is worth considering why the intervention was well-received. The I-SLEEP intervention partners directly with patients to help them improve their own sleep by targeting specific barriers to in-hospital sleep (i.e., noise, light, nighttime disruptions) that were previously identified by patient focus groups [20] and are in line with sleep barriers identified in other studies [30, 31]. Placing patients at the center of the intervention’s development, execution, and desired outcomes likely played a major role in I-SLEEP’s feasibility and acceptability. In terms of I-SLEEP improving sleep outcomes among hospitalized patients, past research has found that higher perceived control over sleep among general medicine inpatients was associated with longer sleep duration and better sleep quality [12]. Therefore, I-SLEEP may be directly linked to empowering patients to improve their sleep through reducing vital signs disruptions. It is also possible that patients with a high degree of empowerment were more likely to use the I-SLEEP questions.

In preparing for a future randomized controlled trial, it is also important to understand why some parts of the intervention, such as the ear plugs and eye masks, were not utilized as much by patients. Given that 78% of patients who used the eye masks were satisfied with them, the low uptake of eye masks may be due to patients simply forgetting they were available to them during their hospitalization. Therefore, in order to increase the impact of the I-SLEEP intervention, educating and encouraging nurses to remind patients about the availability of eye masks may be beneficial. With respect to the ear plugs, given that only three patients used them and only one patient (33%) was satisfied with them, the ear plugs may not have been used due to either low perceived benefit or a comfort or effectiveness issue. Other studies, for example, have documented low usage of ear plugs in hospitalized patients because they were not user-friendly or noise levels were not high enough to warrant them [32, 33]. In order to make the I-SLEEP intervention as impactful and cost-effective as possible, it is worth considering changing ear plug manufacturers to find a more comfortable model or removing the ear plugs from the intervention kit entirely.

This study’s limitations should be acknowledged. First, this is a single-site pilot study with a small sample size, which limits how generalizable the results may be regarding the intervention’s feasibility and acceptance. Second, this study’s self-reported data could have been affected by external confounding factors (i.e., patient health, energy status, prognosis). While this pilot study also lacks actigraphy data and long-term outcomes that would provide an objective sustained evaluation of I-SLEEP’s effect on sleep both in the hospital and after discharge, a future randomized controlled trial will include objective sleep outcomes as well as longer-term follow-up.

## Conclusion

Hospitalized patients experience numerous nighttime disruptions that prevent them from getting much-needed rest. This study demonstrates that a brief, patient-centered education and empowerment intervention to improve hospitalized patients’ knowledge and empowerment surrounding inpatient sleep (I-SLEEP) is acceptable to patients and can be effectively adopted in a busy tertiary care center to reduce unnecessary nighttime disruptions for patients. This study demonstrated that I-SLEEP was both well-accepted by patients and improved patients’ knowledge and empowerment surrounding sleep during hospitalization. Indeed, this study’s findings suggest that a refined I-SLEEP intervention is ready to be evaluated against standard care in a future randomized controlled trial.

## Data Availability

The datasets used and/or analyzed during the current study are available from the corresponding author upon reasonable request.
